# The Application Potential and Future Challenges of Plant-Derived Exosome-like Nanoparticles in the Treatment of Liver Diseases

**DOI:** 10.3390/pharmaceutics18101315

**Published:** 2026-10-16

**Authors:** Li Ye, Zhichao Fan, Xin Zhang, Yanrong Chen, Jie Zhang, Shaowei Xie, Tao Yu, Hua Yu

**Affiliations:** 1 School of Medical and Life Sciences, Chengdu University of Traditional Chinese Medicine, Chengdu 610075, China; yeli@stu.cdutcm.edu.cn (L.Y.); zhangxin1@stu.cdutcm.edu.cn (X.Z.); zjieson1937@stu.cdutcm.edu.cn (J.Z.); 2 Liver Transplant Center, Organ Transplant Center, Department of General Surgery, West China Hospital of Sichuan University, Chengdu 610041, China; fan-zhi-chao@163.com; 3 Department of Oncology, Southwest Hospital, Army Medical University, Chongqing 400038, China; cqchenyr@163.com; 4 School of Basic Medical Sciences, Chengdu University of Traditional Chinese Medicine, Chengdu 610075, China; 18876370719@163.com; 5 Department of Cardiac Surgery, Sichuan Provincial People’s Hospital, University of Electronic Science and Technology of China, Chengdu 610072, China; 6 Department of General Surgery, Hospital of Chengdu University of Traditional Chinese Medicine, Chengdu 610072, China

**Keywords:** exosome, plant-derived exosome-like nanoparticles, nanomedicine delivery, biotherapy, liver diseases

## Abstract

Liver disease is a serious global health challenge, with high morbidity and mortality that put significant socioeconomic stress on society. Despite therapeutic advances, current regimens have been limited by rapid drug resistance and insufficient targeting accuracy, and there is an urgent need for new methods. Plant-derived exosome-like nanoparticles (PELNs) are a promising natural nanotherapeutic platform due to their low immunogenicity, high biocompatibility, and prolonged stability in biological fluids. We review the biological sources, basic properties, and key preparative techniques for PELNs and characterize their mechanisms of action and therapeutic use in common liver diseases. We analyze their multiple-target hepatoprotective mechanisms including liver-specific targeting pathways and reducing critical inflammatory and antioxidant cascades. We summarize the therapeutic performance of different PELNs in major hepatic models including hepatocellular carcinoma, hepatic fibrosis, alcohol-related liver disease, and acute liver failure, highlighting key plant sources, primary biological effects, and precise molecular pathways. Moreover, one of the major methodological contributions of this work is the proposal of a new recommended extraction framework based on differential and density gradient centrifugation, optimized specifically for different plant tissue types (leaves, roots/stems, and fruits) to minimize technical heterogeneity. Finally, we discuss the major challenges that impede clinical translation of these nanovesicles, including incompletely elucidated mechanisms, imperfect manufacturing practices, and inadequate safety evaluation mechanisms, pointing to important, useful directions for future applications in hepatology.

## 1. Introduction

Liver diseases, including hepatocellular carcinoma (HCC), hepatic fibrosis (HF), acute liver failure (ALF), metabolic dysfunction-associated fatty liver disease (MAFLD), alcohol-related liver disease (ALD), and viral hepatitis (VH), are major global health burdens. Their high morbidity and mortality rates impose substantial socioeconomic pressure. It is estimated that in 2021, 529,202 new cases of liver cancer and 483,875 related deaths have been reported worldwide [1]. In advanced HCC, surgical intervention is often precluded, and the effectiveness of targeted treatments (e.g., lenvatinib) or immune checkpoint inhibitors (ICIs), is often challenged by fast drug resistance due to tumor microenvironment remodeling [2,3]. Also, chronic conditions such as HF lack effective treatments that target pathogenic cells, especially activated hepatic stellate cells [4]. This lack of effective treatment highlights the need for new safe and effective treatments.

These issues have motivated interest in extracellular vesicles (EVs), particularly exosomes, which possess the potential for their ability to transport signaling proteins, nucleic acids, and bioactive molecules across biological barriers [5]. Exosome concentration, morphology, and composition are closely associated with the state of the organism and have been implicated in a number of diseases, such as cardiovascular diseases [6], neurodegenerative diseases [7], and cancer [8]. There are no exosome-based therapeutics; however, as of 2025, more than 200 clinical trials involving exosomes have been performed worldwide for cancer, cardiovascular diseases, infectious diseases, and metabolic disorders [9]. Mammalian-derived exosomes, e.g., mesenchymal stem cell-derived exosomes (MSC-Ex), have been shown to have considerable hepatoprotective ability [10]. Much research has shown that the use of MSC-Ex can inhibit the activation and proliferation of HSCs and promote a microenvironment with favorable qualities for hepatocyte regeneration and functional recovery. They mitigate oxidative stress by delivering glutathione peroxidase 1 (GPX1) and regulate apoptosis via ERK1/2 and NF-κB signaling pathways [11]. However, their broad clinical translation is severely hindered by mass production difficulties, high costs, and potential immunogenicity.

Plant-derived exosome-like nanoparticles (PELNs) are an attractive alternative to these translational hurdles [12]. Research on PELNs can be traced back to 1967, when Walter Halperin and colleagues first observed exosome-like structures in carrot cells by transmission electron microscopy (TEM) [13]. It was not until 2013 that Songwen Ju et al. extracted and described PELNs from grapes, showing that they are absorbed by the mammalian gut and they are involved in modulating inflammatory responses [14]. It has been shown in preclinical studies that PELNs have remarkably low immunogenicity in the human body, remain stable in biological fluids, and thus have therapeutic effects [12,15]. PELNs from ginger, ginseng, aloe vera and lemon are found to reduce inflammatory bowel disease (IBD) by modifying immune response, repairing the intestinal epithelium barrier, and regulating the gut microbiota [16]. Several studies have successfully loaded therapeutic agents into PELNs [17]. A core–shell biomimetic nanocarrier (CEXO@ZIF-8/DOX) was developed by covering a doxorubicin-loaded zeolitic imidazolate framework-8 (ZIF-8) core within a *Cucurbita moschata*-PELNs shell [18]. Given the liver’s complex microenvironment and chronic inflammation conditions, this combination of endogenous bioactivity and engineered targetability makes PELNs very promising candidates for liver disease.

Notably, a universally accepted standard protocol for the isolation and purification of PELNs has yet to be established. However, the lack of standard isolation protocols, coupled with plant tissue rigidity and inter-species heterogeneity, also limits data reproducibility and compromises vesicular yield and purity. Currently, research on PELNs is progressively transitioning from preliminary phenotypic characterization toward rigorous mechanistic exploration. Consequently, elucidating their biological characteristics, specific mechanisms of action, and disease-specific therapeutic potential has emerged as a central focus for the next phase of this field. In order to address these issues, we systematically describe biological sources and basic features of PELNs, discuss key preparative technologies, propose a standardized plant tissue-based extraction strategy based on the literature, and discuss their applications and potentials in liver disease therapeutics by assessing their intrinsic biological effects as well as their potential as targeted delivery systems, as a reference for this research field.

### 1.1. Characteristics and Biogenesis of PELNs

#### 1.1.1. Classification and Nomenclature of PELNs

PELNs can be classified based on various characteristics, including their source and methods of isolation and purification. Depending on their plant source, PELNs can be categorized into those derived from fruits, vegetables, and medicinal plants, among others [19]. In addition, engineered PELNs have emerged as a popular area of research in recent years; through engineering approaches such as artificial modification, these PELNs are endowed with various biological functions, thereby distinguishing them from natural PELNs [20].

Currently, some researchers have proposed classifying and naming these vesicles based on their isolation and purification methods. For example, the term “plant extracellular vesicles (PEVs)” is used to describe vesicles isolated from the extracellular environment of plant cells, such as leaf exudates or root secretions, through tissue homogenization. These PEVs typically originate from the plasma membrane or the endosomal system and exhibit classic exosome characteristics; In contrast, “plant-derived nanovesicles” (PDNVs) refer to the entire population of vesicles extracted via destructive methods such as tissue homogenization. It remains unclear whether these vesicles are naturally released into the extracellular space under physiological conditions; this population may include intracellular endosomes as well as vesicles formed by the reorganization and fusion of damaged cell membranes during the extraction process [21]. Within the category of PDNVs, some researchers have established further subcategories. Some researchers regard PELNs as primary products obtained from disrupted plant tissue (such as freshly extracted juice) processed using high-speed homogenizers or stirrers, while those subjected to further purification are termed plant lipid-derived nanovesicles (PLNVs) [22].

Given that a unified nomenclature has not yet been established, most of the literature reviewed in this paper tends to use the term “PELNs”. The term “exosome-like” is used solely to describe the nanoscale morphology, membrane-enclosed structure, and cargo-carrying capacity of PELNs, which are similar to those of exosomes, rather than to imply that they are identical to mammalian exosomes in terms of biogenesis or marker identity [23]. Therefore, when discussing the applications and prospects of such plant-derived nanovesicles in the treatment of liver diseases, this paper will primarily focus on PELNs and the PLNVs obtained through their further purification.

Furthermore, to eliminate the confusion caused by the diverse and inconsistent abbreviations used in various original studies, this work adopts a standardized nomenclature format, “Plant source + PELNs,” throughout the text.

#### 1.1.2. Characterization of PELNs

• Physical Properties of PELNs

PELNs are primarily characterized by their morphology, particle size distribution, and zeta potential. In TEM, PELNs typically appear as spherical or ellipsoidal vesicular structures. In contrast, cryo-electron microscopy (cryo-EM), which preserves a more native state, has confirmed that these structures are predominantly regular spheres [24,25,26]. The size of PELNs is still controversial: in some studies based on a large number of isolated samples, they reported a wide particle size distribution ranging from 30 nm to 1000 nm [27,28]. Other studies tend to define a narrower range of 50–500 nm [29], closer to the normal size of animal-derived exosomes. Geometric variation is strongly affected by plant taxonomy and processing. The particle size of PELNs varies substantially, highly confounded by plant species, tissue origin, and growth conditions, as well as by isolation and characterization.

**Figure 1:**
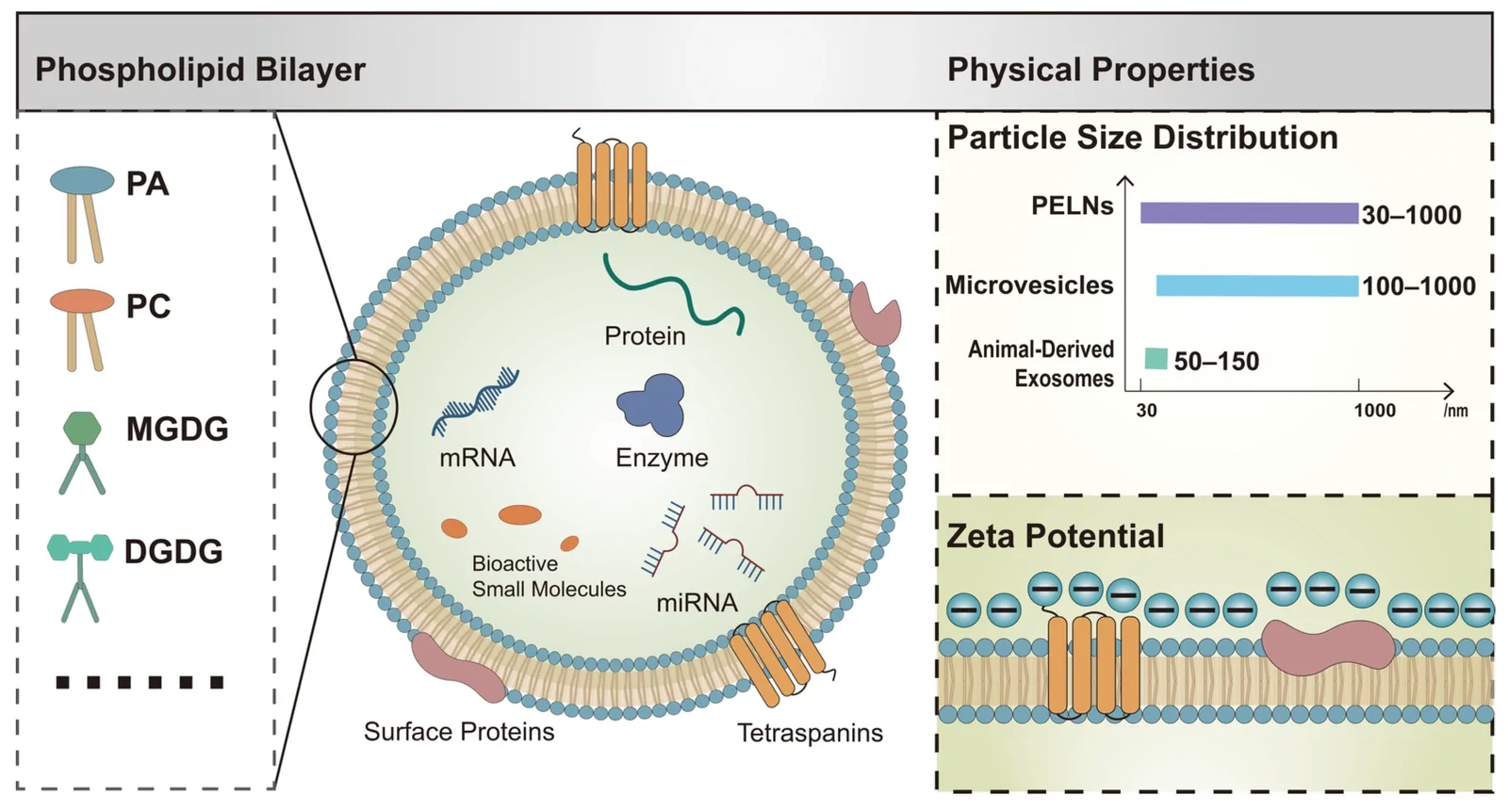
The diagram illustrates the physical morphology of PELNs, along with their characteristic lipid bilayer architecture. It reveals an enrichment of lipid components such as phosphatidic acid (PA) and phosphatidylcholine (PC), as well as the encapsulated contents comprising miRNAs, proteins, and small-molecule bioactive metabolites. As the composition and properties of PELNs vary considerably depending on the plant source, tissue type, growth conditions, and isolation methods, it is worth noting that the structure shown here is merely a generalized schematic and does not represent a universal standard.

Zeta potential is one of the most important parameters for evaluating colloidal stability and surface electrochemical properties of nanoparticles. By measuring the net surface charge, it determines directly the electrostatic repulsion and aggregation tendency of particles in a dispersion system [30]. Numerous studies have demonstrated that, because the membrane structure of PELNs is rich in lipid bilayers and surface proteins (e.g., transmembrane proteins), these structures typically carry a net negative charge at physiological pH. The zeta potential of PELNs is not static but varies with environmental conditions. A study of *Ginger*-PELNs showed that in very acidic environments (like gastric fluid), PELNs can carry a weak positive charge (+0.26 mV) [31]. Figure 1 depicts the key physicochemical properties of PELNs.

• Chemical Composition of PELNs

At the molecular level, PELNs come in various species of lipids, proteins, nucleic acids, and small-molecule metabolites [19]. This is the reason PELNs can bind across kingdoms and, in turn, mediate intercellular communication.

The lipid bilayer is highly enriched in plant-specific phospholipids and galactolipids, such as phosphatidic acid (PA), phosphatidylcholine (PC), digalactosyldiacylglycerol (DGDG), and monogalactosyldiacylglycerol (MGDG) [32]. In addition, cholesterol, a hallmark component of animal-derived exosomes, has not been detected in PELN membranes to date. This plant-specific lipidomic signature fundamentally shapes vesicle biophysics, providing structural protection for encapsulated payloads while governing membrane fusion kinetics to facilitate cross-kingdom cellular uptake and intrinsic targeting. The PELN lipidomic profile also shows the presence of significant species-dependent heterogeneity. The fatty acid content of *Ginger*-PELNs was nearly 40 times higher than that of *Grape*-PELNs and 5.6 times higher than that of *Grapefruit*-PELNs [33]. These profound compositional distinctions suggest that different plant-derived PELNs may have different physicochemical properties and possible biological functions.

Besides their rich lipid content, PELNs carry several nucleic acids, mainly RNA (mRNA, miRNA, siRNA, other non-coding RNAs, and small amounts of DNA fragments). Small RNAs such as miRNAs have been demonstrated to possess cross-kingdom regulatory capabilities. *Polygonum multiflorum*-PELNs contain various miRNAs, such as aof-miR168a and osa-miR164a [34].

Comprehensive proteomic profiling has revealed that PELNs possess a highly complex and functionally diverse proteome, responsible for transmembrane transport, stress adaptation, and cross-kingdom immunomodulation. A total of 1843 proteins have been found in *Catharanthus*-PELNs that are enriched with transporter and signaling proteins, including members of the ATP-binding cassette (ABC) transporter family, enzymes, and endosomal membrane proteins [35]. Although advanced proteomic profiling has successfully mapped the extensive protein complexity of PELNs, establishing universally accepted pan-plant vesicle biomarkers remains a long-standing challenge due to the difficulty in isolating PELNs imposed by the plant cell wall, the unique nature of plant unconventional secretory pathways, and the fact that PELNs exhibit pronounced proteomic heterogeneity across diverse plant species. Recent studies have found a number of potential marker proteins in *Arabidopsis thaliana*, namely tetraspanin 8 (TET8) and tetraspanin 9 (TET9) that are homologous to animal exosome marker CD63, as well as the PEN1 protein from the t-SNARE superfamily [36,37].

Aside from macromolecular payloads, PELNs selectively enrich a species-specific repertoire of pharmacologically active secondary metabolites including curcuminoids [38,39], carotenoids [40], and flavonoids [41,42,43,44]. These metabolites are integral to the distinctive biological functions of PELNs (Table 1).

### 1.2. Biogenesis of PELNs

**Figure 2:**
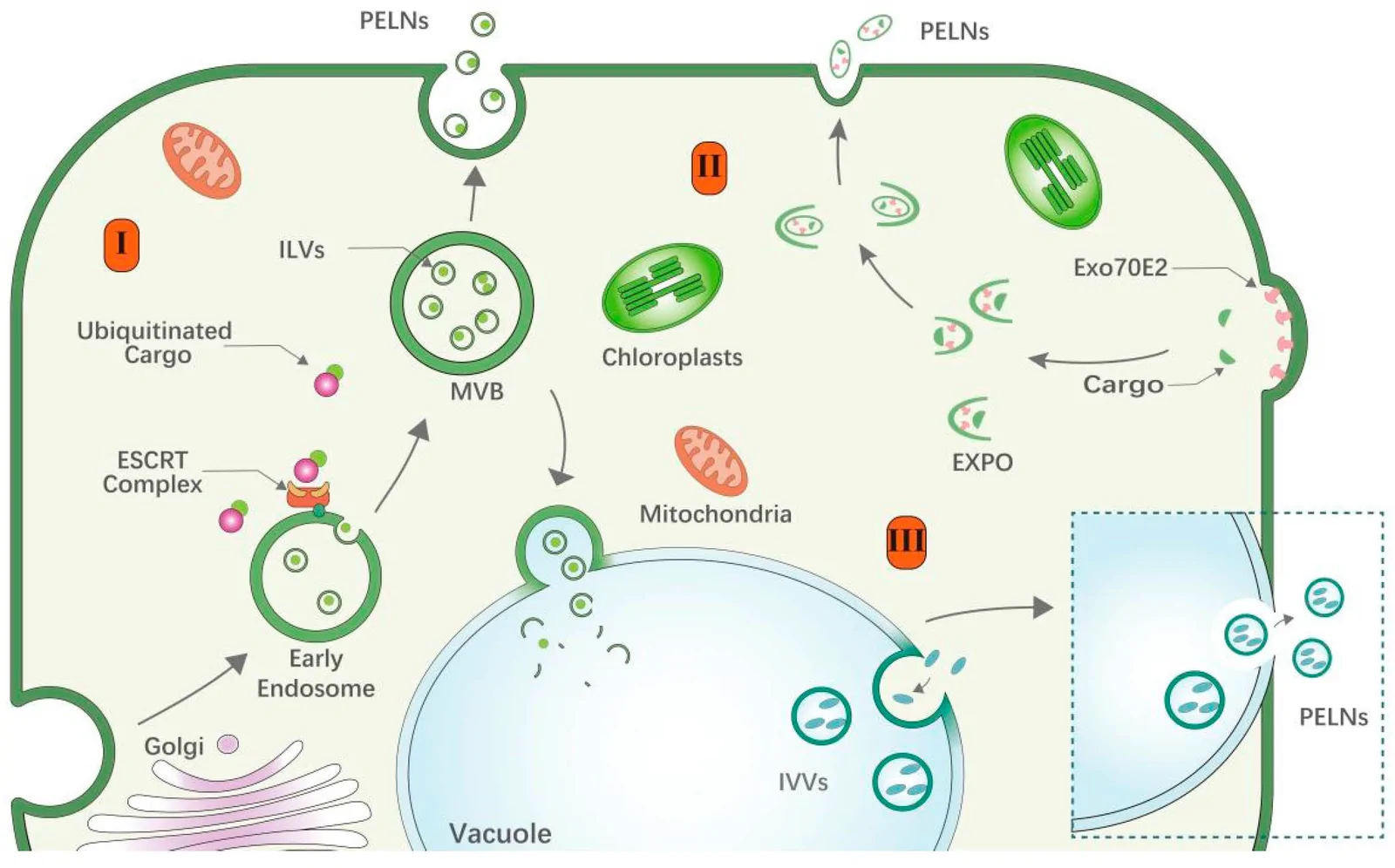
Proposed biogenesis pathways for specific subpopulations of plant extracellular vesicles, based on the current literature. The diagram illustrates three postulated routes: I. the classical MVB pathway; II. the EXPO pathway; and III. the vacuolar pathway. Notably, the MVB pathway involves the participation of the ESCRT. ILVs: intraluminal vesicles; IVVs: intravacuolar vesicles.

Currently, based on studies in model plants, their biogenesis is thought to primarily involve three pathways: the classical multivesicular body (MVB) pathway, the plant-specific exocyst-positive organelle (EXPO) pathway, and the vacuolar pathway [56] (Figure 2).

However, a strict distinction must be drawn between the established secretory mechanisms of intact cells and the speculative origins of PELNs. Because current extraction protocols rely heavily on aggressive mechanical disruption, such as grinding, blending, juicing, or high-pressure homogenization, the recovered isolates inevitably comprise a heterogeneous mixture of true extracellular vesicles and artificial membrane vesicles derived from fragmented intracellular organelles [57]. Consequently, attributing these natural biogenesis pathways directly to all particles recovered post-extraction remains highly speculative. In light of this fundamental limitation, the mechanisms illustrated in Figure 2 should be strictly interpreted as “putative biogenesis pathways for specific plant extracellular vesicle populations” rather than the definitive source for all particles currently classified as PELNs.

#### 1.2.1. MVB Pathway

The MVB pathway is the most studied biogenesis pathway of PELNs and is analogous to the production of mammalian exosomes. The pathway starts by endocytosis of the plasma membrane, leading to early endosomes that mature into late endosomes or MVBs by invagination of the limiting membrane, thereby producing intraluminal vesicles (ILVs) [58]. It is closely related to the trans-Golgi network (TGN) and depends on both the endosomal sorting complex required for transport (ESCRT) and ESCRT-independent mechanisms such as sphingolipid metabolism [59]. Later on, mature MVBs either undergo vacuolar fusion to degrade content or migrate along the cytoskeletal network to fuse with the plasma membrane and release ILVs as PELNs into the extracellular space.

The molecular mechanisms governing the targeted fusion of MVBs with the plasma membrane represent a current research frontier and a significant challenge. Recent studies have provided important insights into this process. During pathogen infection, MVBs that normally traffic to the vacuole rewire their path, are recruited to the infection site, and release exosomes containing defense material [60]. Tao et al. reported that plasma membrane-localized ARM-type E3 ubiquitin ligases SAUL1 and AtPUB43 control anchoring of MVBs to the plasma membrane during Phytophthora capsici infection of Nicotiana benthamiana [61].

#### 1.2.2. Other Biogenesis Pathways

Besides the classical MVB pathway, plants may also utilize alternative routes for the generation of EVs. The EXPO pathway is a plant-specific non-conventional secretory pathway [62]. EXPO is a double-membrane-bound organelle that is independent of autophagic signaling. It was shown that the outer membrane of EXPO can fuse with the plasma membrane to release its internal single-membrane vesicles to the cell wall space, resulting in the formation of PELNs [63]. Another possibility, where MVBs or their ILVs may be discharged from the cell through an indirect, vacuole-mediated mechanism, is also possible; however, their precise molecular machinery remains incompletely understood [64].

## 2. Extraction of PELNs

Although research on PELNs has entered a phase of rapid development, the standardization of its methodologies lags significantly behind that of animal exosomes. The unique architecture of plant cell walls and the complexity of their secondary metabolites pose intrinsic obstacles to standardizing extraction protocols. Currently, various isolation methods (e.g., ultracentrifugation, size exclusion chromatography, and polymer precipitation) and purification strategies (e.g., density gradient centrifugation, ultrafiltration, and affinity capture) are widely employed in the field. Each of these methods has its merits and drawbacks in terms of extraction efficiency, vesicle integrity, cargo capacity, and operational feasibility [65]. Thus, the selection of a protocol must take into account plant source, downstream applications, and experimental conditions.

### 2.1. Plant Pre-Treatment

The selection of an appropriate plant pre-treatment method is a critical step in enhancing the yield and purity of PELNs. Depending on the plant material, common pre-treatment methods can be grouped as tissue disruption and infiltration centrifugation [65,66]. In addition, to meet specific requirements, a number of techniques such as freeze–thaw cycling, high-pressure treatment, and enzymatic treatment have been developed.

Mechanical disruption physically releases plant sap and is generally applicable to parenchymal tissues with fragile cell walls and high moisture content, such as those found in fruits, stems, and leaves [67]. Some researchers use liquid nitrogen to render the plant tissue brittle and powdery, followed by grinding it into a fine powder under low-temperature conditions to avoid heat-induced degradation or aggregation of the exosome-like nanoparticles, which is referred to as the liquid nitrogen grinding method [68]. Although this compromises cellular integrity, leading to extracts contaminated with organelle fragments and ribosomes and thereby complicating downstream purification, its operational simplicity and high throughput have made it the most widely adopted method.

For plant tissues with low water content and tough texture—for example, root systems—that are difficult to process effectively using mechanical disruption methods, tissue infiltration extraction is usually performed, where the specific isolation buffer is added to the plant tissue, vacuum equipment is used to facilitate thorough infiltration of the liquid, and the extracted plant sap is collected by low-speed centrifugation [69]. Compared with mechanical disruption methods, it better preserves the structural integrity of vesicles and effectively reduces interference from cellular debris and tissue residues. However, its product yield is relatively low, and the operational procedure is complex.

Building on these methods, a series of improved techniques aimed at enhancing selective release efficiency have been successively developed. Enzymatic pre-treatment employs pectinase and cellulase to hydrolyze plant cell walls, thereby gently dissociating the tissue. For instance, Xin Han et al. incubated *Ginkgo biloba* leaves with cellulase (2–4% *v*/*v*) and pectinase (0.25–0.5% *v*/*v*) for 3–6 h to release protoplasts [70]. This approach effectively preserves vesicle integrity yet it is limited by lengthy incubation, high enzyme costs, and possible interference from residual enzymes. High-pressure homogenization employs dynamic high pressure to force samples through a narrow slit valve, generating intense shear forces, cavitation effects, and turbulence to efficiently disrupt plant cell walls [71]. Characterized by high disruption efficiency and speed, this technique is suitable for the rapid preparation of large batches of samples. The freeze–thaw cycle lyses cells by shear forces from ice crystals in repeated freeze–thaw cycles. Although the freeze–thaw cycling method (typically alternating between −80 °C and room temperature) is widely adopted because it requires no specialized equipment, it introduces severe physical and thermal stresses. Therefore, the number of cycles and temperature parameters must be rigorously optimized to ensure the yield and structural integrity of PELNs [72].

### 2.2. Isolation Techniques for PELNs

After the extraction of crude plant extracts, suitable methods should be used to extract and enrich vesicles. Existing methods may be based on the physical properties of vesicles (sedimentation coefficient, particle size, density, etc.) or on the biological aspects of vesicles. A comprehensive comparison of these techniques is provided in Table 2.

#### 2.2.1. Ultracentrifugation Techniques

Differential ultracentrifugation (dUC) removes dead cells, large debris, and organelles sequentially by gradually increasing the centrifugal force and finally settles vesicles at ≥100,000× *g* [73]. The typical process includes removal of cells and large debris at 300–500× *g*; removal of apoptotic bodies and large vesicles at 2000–10,000× *g*; removal of smaller organelle fragments at 10,000–20,000× *g*; and finally, deposition of vesicles at 100,000× *g* for 60–90 min to enrich PELNs. Some studies have resuspended the pellet in buffer and then subjected it to ultracentrifugation again in order to further purify the product and remove contaminating proteins.

For purity, density gradient ultracentrifugation (DGUC) using sucrose or iodixanol gradients allows nanoparticles to be distributed into their isopycnic regions within 12–18 h, enabling the precise isolation of highly purified PELNs within the specific density range of 1.10–1.19 g/mL. Kwangsun Seo et al. successfully extracted high-purity PELNs from ginseng by sucrose density gradient centrifugation [46].

#### 2.2.2. Ultrafiltration Techniques

Ultrafiltration (UF) separates molecules according to size using semipermeable membranes that retain larger molecules, such as cell debris, and allow small molecules to pass through. In practice, it is possible to use pressure-driven ultrafiltration and centrifugal ultrafiltration to improve filtration efficiency and yield. Kartik Kumar Sarwareddy et al. extracted *Tomato*-PELNs via centrifugation and ultrafiltration [74]. Yunyi Chen et al. combined differential centrifugation, sucrose gradient density centrifuging, and ultrafiltration to extract PELNs from over ten plant species [75].

Although ultrafiltration is rapid and easily scalable for large volumes, its size exclusion principle inevitably co-retains similarly sized contaminants, such as viral particles and protein aggregates. Consequently, it often suffers from membrane fouling and is typically deployed for initial volume reduction rather than as a standalone purification step.

#### 2.2.3. Size Exclusion Chromatography

Size exclusion chromatography (SEC) uses porous gel beads to separate particles by their hydrodynamic volume; larger particles bypass the pores and are eluted earlier than smaller particles trapped within the pores. By avoiding the intense shear forces inherent to ultracentrifugation, this physical separation effectively preserves the native structure and activity of PELNs. Aleksandra Steć et al. extracted PELNs from mango using differential centrifugation and SEC [76]. Do Kyung Kim extracted PELNs from carrots using ultrafiltration and SEC and characterized them by TEM and nanoparticle tracking analysis (NTA) [77].

Recently, the use of microfluidic chips with SEC (µSEC) allows device miniaturization and automation with significantly lower processing time and potentially clinical real-time detection [66]. For the isolation of animal exosomes, SEC still has considerable room for improvement regarding the co-separation of lipoproteins [78], and this must be considered when applying SEC to PELN extraction.

#### 2.2.4. Polymer Precipitation Method

The polymer precipitation method is attractive for large-scale preparation due to its operational simplicity, high throughput, and low equipment requirements. In studies involving the extraction of exosomes from animal sources, the polymer precipitation method may be superior to ultracentrifugation in maintaining the structural integrity and biological activity of exosomes [79]. The principle involves the use of hydrophilic polymers, such as polyethylene glycol (PEG), to sequester the hydration shell surrounding the vesicles, thereby reducing their solubility and inducing aggregation and precipitation [80]. A typical PEG precipitation method employs PEG with a molecular weight of 6000–20,000 Da, followed by low-speed centrifugation after incubation at low temperature to collect the precipitate. Most researchers utilize the polymer precipitation method as a step to enrich exosomes, subsequently combining it with other purification techniques. For instance, Kocholata et al. first employed PEG precipitation to enrich vesicles from crude extracts of tobacco leaves, followed by DGUC to obtain highly purified PELNs [81].

#### 2.2.5. Immunoaffinity Capture

Immunoaffinity capture (IAC) is a highly selective isolation technique that utilizes specific antibodies to recognize and bind to specific antigens on the surface of exosomes. This method involves immobilizing antibodies or affinity ligands that target vesicular surface markers onto a solid-phase carrier, such as magnetic beads, followed by sequential capture, washing, and elution. The key to IAC lies in the selection of specific surface markers. In research on animal exosomes, tetraspanins (such as CD9, CD63, and CD81) are the most commonly used and recognized surface markers; in addition, heat shock proteins (such as HSP70), integrins, and EpCAM are also frequently employed for the capture of exosomes from specific sources [82]. However, in PELNs, no unique surface markers specific to IAC have yet been identified.

Some studies have attempted to capture PELNs using antibodies directed against plant tetraspanins (such as TET8 and TET9); for example, Yifan Huang et al. reported an antibody capable of specifically recognizing the EC2 domain of TET8, which successfully isolated TET8-positive exosomes from *Arabidopsis thaliana* [73]. However, given the high degree of sequence variation across species, the efficiency and broad applicability of these methods remain to be verified. Other studies have focused on non-protein markers; for example, Wataru Nakai utilized the Tim-4 protein to recognize phosphatidylserine (PS) on the surface of EVs, thereby achieving affinity-based separation independent of protein markers [83]. Although these strategies have been successful in animal systems, their direct application to PELNs still poses significant challenges. The core difficulty lies in the lack of a recognized and universally applicable surface antigen specific to PELNs, which severely limits the large-scale application of IAC in this field.

### 2.3. Methodological Comparison and Impact on PELN Properties

Currently, no single separation method can perfectly balance yield, purity, and scalability. Methods that are easy to scale up and offer high yields, such as UF and PEG precipitation, typically co-isolate unwanted non-vesicular macromolecules. In contrast, DGUC and IAC can achieve high purity, but their cumbersome procedures, low recovery rates, and poor scalability make them difficult to apply in large-scale clinical translation. Beyond basic process parameters, the extraction process itself can substantially affect the physicochemical properties of the vesicles. For example, the strong shear forces and ultra-high centrifugal forces involved in dUC can easily disrupt the fragile lipid bilayer, leading to vesicle aggregation or premature release of the therapeutic RNAs and metabolites they encapsulate [84,85].

Chemical separation methods can also introduce artifacts. After PEG precipitation, for instance, a polymer coating typically remains on the vesicle surface [86]. This masking effect may obscure key membrane proteins, consequently compromising their downstream biological functions. IAC faces similar risks, as harsh acidic or high-salt elution buffers can easily denature the surface antigens that mediate cellular interactions [87]. Although SEC effectively avoids the aforementioned physical and chemical damage, its throughput remains limited by the sample volume.

Ultimately, to achieve an optimal balance between yield, purity, and structural integrity, combined extraction strategies, such as UF coupled with SEC, are increasingly accepted as reproducible and scalable approaches, as they minimize the negative impact of any single method on the physicochemical properties of PELNs.

## 3. Recommended Extraction Strategies Based on Plant Tissue Types

The extreme heterogeneity of plant tissues poses a major challenge to standardization. From soft, succulent fruits to hard, lignified stems, and from thin-walled leaves to fiber-rich roots and rhizomes, different tissues exhibit significant variations in cell wall structure, degree of lignification, sap content, and the composition of secondary metabolites (such as polyphenols, starch, and mucilage). Although a variety of extraction methods have been reported, it is often difficult to apply a single, universal protocol to all plant materials.

**Figure 3:**
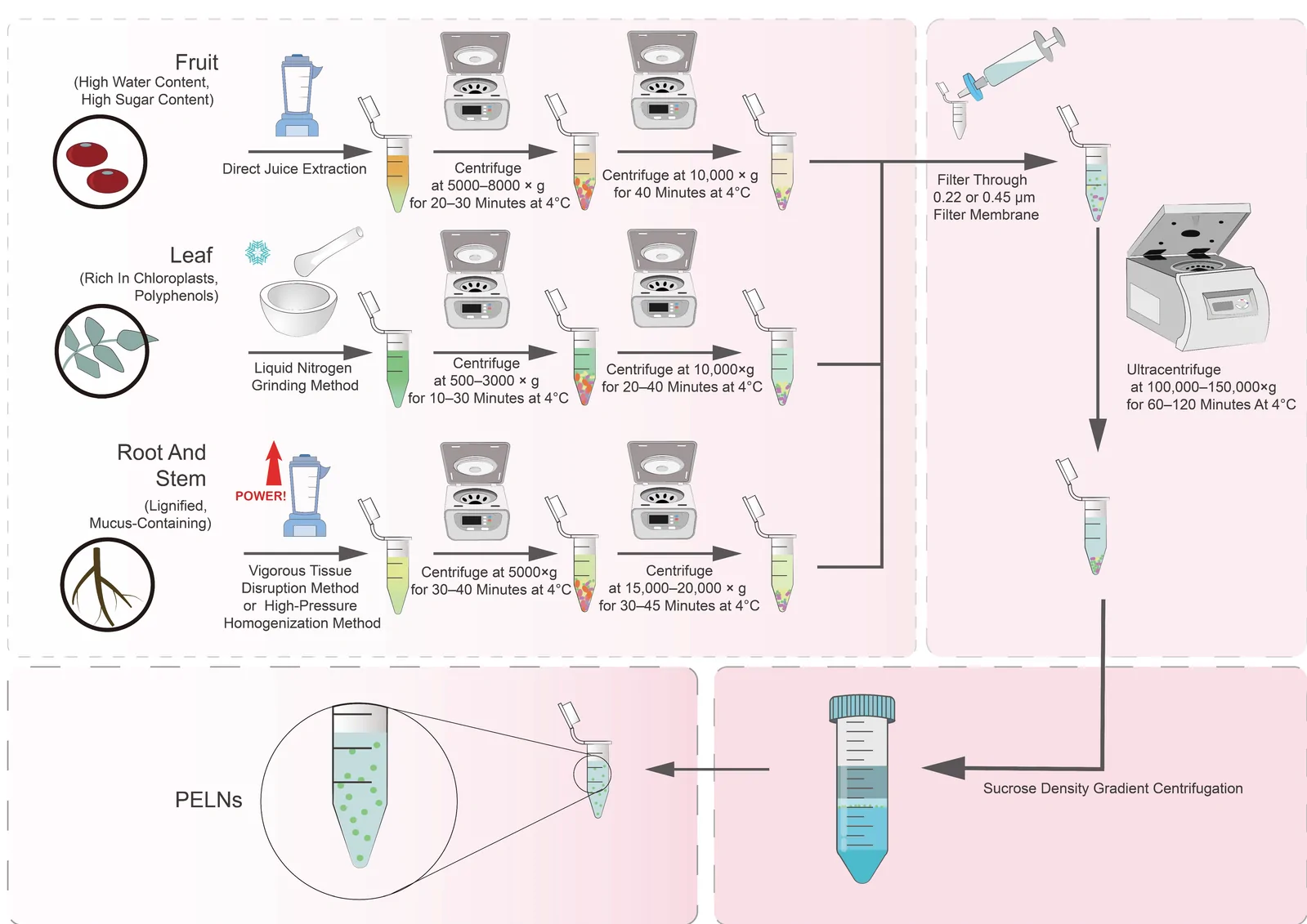
Flowchart of recommended extraction strategies based on plant tissue types. This flowchart compares recommended extraction strategies for three types of plant tissues: leaves, roots/stems, and fruits. It details the entire process from tissue pre-treatment and differential centrifugation enrichment to density gradient centrifugation purification.

Accordingly, rather than proposing a definitive protocol, we outline these recommended extraction strategies by synthesizing the strengths and limitations of current methodologies. Because some parameters are adapted from related botanical techniques, this provisional, tissue-informed framework requires empirical validation. We expect future studies to build upon this baseline by evaluating measurable criteria (such as particle recovery, particle-to-protein ratio, membrane integrity, organelle contamination, and bioactivity retention) to ultimately achieve data-driven standardization (Figure 3).

### 3.1. Impact of Inter-Tissue Differences on PELNs Extraction

The extraction efficiency and purity of PELNs are profoundly influenced by the physicochemical properties of the source tissue.

In lignified tissues such as mature stems and root bark, lignin is also deposited to form a strong secondary cell wall [88]. Differences in plant cell walls are factors that determine the optimal mechanism of tissue disruption. Non-lignified tissue such as young leaves and fruit pulp can be strongly disrupted by gentle mechanical homogenization. However, highly lignifying tissues strengthened by strong secondary wall structures such as matured stems, roots, and bark require more rigorous physical disruption or digestion through cellulase and pectinase.

Furthermore, sap composition affects the hydrodynamics of the original homogenate. Succulent fruits facilitate vesicle release but contain high amounts of sugars, organic acids, and high-viscosity polysaccharides, which obstruct ultracentrifugal sedimentation, and are capable of macromolecular co-precipitation and interfering with downstream purification. Low-moisture woody stems need large buffers, which degrade PELN concentrations and may also have external interference.

### 3.2. Recommended Extraction Strategies

Supported by the current literature, this reference extraction protocol is divided into three stages: (1) tissue pre-treatment, (2) differential centrifugation and density gradient purification, and (3) special protocol optimizations for different tissue types.

#### 3.2.1. Leaf Tissues

Leaf tissue has relatively homogeneous thin cell walls. Chloroplasts, polyphenolic compounds, and waxy cuticles are the main sources of contaminants. Liquid nitrogen grinding is an effective homogenization method. By immediately freezing the tissue, this technique effectively inhibits enzymes like polyphenol oxidase, thereby preventing target molecule degradation and ensuring a full cell wall break. This low-temperature disruption strategy draws on established methods for isolating leaf cell nuclei [89], since the isolation of PELNs and that of leaf cell nuclei both face similar challenges, such as enzymatic degradation and cell wall rigidity. On softer leaves, ice bath homogenization could be implemented: tissues are mixed with pre-cooled PBS or Tris-HCl (1:3 and 1:5 *w*/*v*) and briefly homogenized using a high-speed homogenizer, in 30 s bursts, repeated 2–3 times in a row to prevent excessive heat.

The extract strictly relies on sequential differential centrifugation at 4 °C. A low-speed centrifugation (500–3000× *g*, 10–30 min) removes unbroken tissue pieces, clumps of cells, and large vascular bundle elements. An intermediate centrifugation (10,000× *g*, 20–40 min) reduces leaf-specific organelle contaminants, such as chloroplasts and mitochondria, nuclear debris, and larger microvesicles, such as apoptotic bodies. The recovered supernatant is filtered (0.22 or 0.45 μm) for residual fibers. PELNs are then pelleted by ultracentrifugation (100,000–150,000× *g*, 60–120 min, 4 °C). The resulting pellet is gently resuspended in a small volume (1/100 to 1/1000 of the original volume) of pre-conditioned PBS/buffer using wide-bore pipette tips in order to reduce shear stress-induced vesicle damage.

In order to reach ultimate purity, we start by separating the PELNs from co-precipitated protein aggregates, lipoprotein particles, and ribosomes with discontinuous sucrose density gradient centrifugation. The vesicle suspension is layered onto a gradient composed of 8%, 30%, 45%, and 60% (*w*/*v*) sucrose (20 mM Tris-HCl, pH 7.4) and ultracentrifuged at 100,000–200,000× *g* for 16–18 h at 4 °C. Highly purified PELNs arrive at their isopycnic density of 1.10–1.19 g/mL between 30% and 45% sucrose layers, are recovered, diluted widely with PBS, and cleaned at 100,000× *g* for 70 min to remove residual sucrose and generate highly purified leaf PELNs that can be applied downstream.

#### 3.2.2. Plant Roots and Stems

As essential organs for nutrient transport and structural support, plant roots and stems possess complex structures with variable degrees of lignification and specialized secretions (e.g., mucilage and resin), which in turn dictate their specific extraction requirements. While juvenile roots and stems can be processed via liquid nitrogen grinding analogous to leaf protocols, mature, highly lignified tissues necessitate robust mechanical disruption or high-pressure homogenization. Moreover, tissues rich in mucilage or resin yield exceptionally viscous homogenates. Effectively processing these samples requires increasing the buffer dilution ratio (up to 1:10 *w*/*v*) and incorporating a mucolytic agent (e.g., dithiothreitol, DTT). Alternatively, slightly acidic buffers can be utilized to selectively reduce mucilage solubility, thereby facilitating its efficient removal by centrifugation.

The abundant fibrous and lignified residues present in root and stem homogenates necessitate modifications to the conventional centrifugation protocols. Specifically, the initial centrifugation time must be extended (5000× *g* for 30–40 min) to ensure adequate removal of coarse debris. For tissues rich in starch granules (e.g., tuberous roots) or lipid bodies, the standard intermediate centrifugation at 10,000× *g* is insufficient. Instead, the centrifugation parameters must be intensified to 15,000–20,000× *g* for 30–45 min to successfully sediment these high-density organelles. During the final ultracentrifugation step, to counteract the potentially elevated viscosity inherent in these extracts, the centrifugation time must be prolonged to 90–120 min, or the centrifugal force appropriately increased to 150,000–200,000× *g* to ensure adequate sedimentation of the vesicles.

For root and stem extracts, in addition to sucrose density gradient centrifugation, SEC is particularly useful, as it can effectively remove macromolecular impurities such as polysaccharides and mucilage that may be present in the solution, based on size rather than density.

#### 3.2.3. Plant Fruits

Plant fruits are often very high in water content. They can be directly squeezed without harsh grinding. Low-speed centrifugation or double-layer gauze filtration is possible for pulp. For thick peels, the pulp and peel may be separated. The extracted juice may contain large amounts of pulp, starch granules, or pectin; initial clarification must be achieved by centrifugation at 5000–8000× *g* for 20–30 min. As soluble proteins and lipoproteins in fruits are very high, sucrose density gradient centrifugation can be performed after ultracentrifugation, which separates PELNs from lipoprotein complexes and glycoproteins.

### 3.3. PELN Characterization and Validation Protocols

Given the Minimal Information for Studies of Extracellular Vesicles (MISEV) guidelines, the isolated PELNs must be characterized with multiple complementary methods in order to confirm their vesicular identity and preparation purity and to ensure reliable functional experiments [90].

For physical characterization, NTA, often considered the gold standard for determining particle concentration, and dynamic light scattering (DLS) are used to assess particle concentration and hydrodynamic size distribution, typically revealing a dominant peak within the 50–200 nm range [91]. However, DLS has inherent limitations when analyzing polydisperse samples. Due to its low resolution and the fact that scattered light intensity is proportional to the sixth power of particle size, this can cause signals from small particles to be masked by the strong scattering signals from large particles [92]. Therefore, comprehensive characterization of PELNs must rely on a combination of multimodal methods. TEM and cryo-EM are also designed to directly visualize the cup-shaped or spherical bilayer membrane; in particular, cryo-EM is optimized for the native hydrated state, resulting in the faithful structural representation. As mentioned earlier, the “cup-shaped” structure observed by TEM is often an artifact induced by dehydration and staining during the preparation process. Zeta potential analysis is also used for dispersion stability, as a stable negative surface charge (below −30 mV) indicates a low probability of aggregation.

Beyond physical metrics, biochemical purity is tested by probing extracts for organelle-specific protein contaminants. Mass spectrometry-based lipidomics could be used to dissect the PELN lipidome, and high-throughput sequencing (e.g., small RNA-seq) or qPCR accurately characterizes nucleic acid contents, notably microRNAs.

## 4. Research Status and Advances of PELNs

Current PELN research is built on a dual perspective, examining these nanoparticles both as functional therapeutics and as natural delivery vehicles. Taken together, these complementary lines of investigation provide the evidence base needed to advance their clinical translation.

### 4.1. Potential as Drug Delivery Systems

Non-specific drug biodistribution resulting in off-target toxicity and low bioavailability remains an important bottleneck in drug development. To address this, drug delivery systems (DDSs) are designed to accurately transport active drugs to the target site, which in turn optimizes their pharmacokinetic and pharmacodynamic profile [93]. While many conventional synthetic nanocarriers, including liposomes, are able to improve water solubility and extend systemic circulation, they are still limited by intrinsic drawbacks such as immunogenic risks, uncontrolled biodistribution resulting from off-target effects, and high manufacturing cost [94].

**Figure 4:**
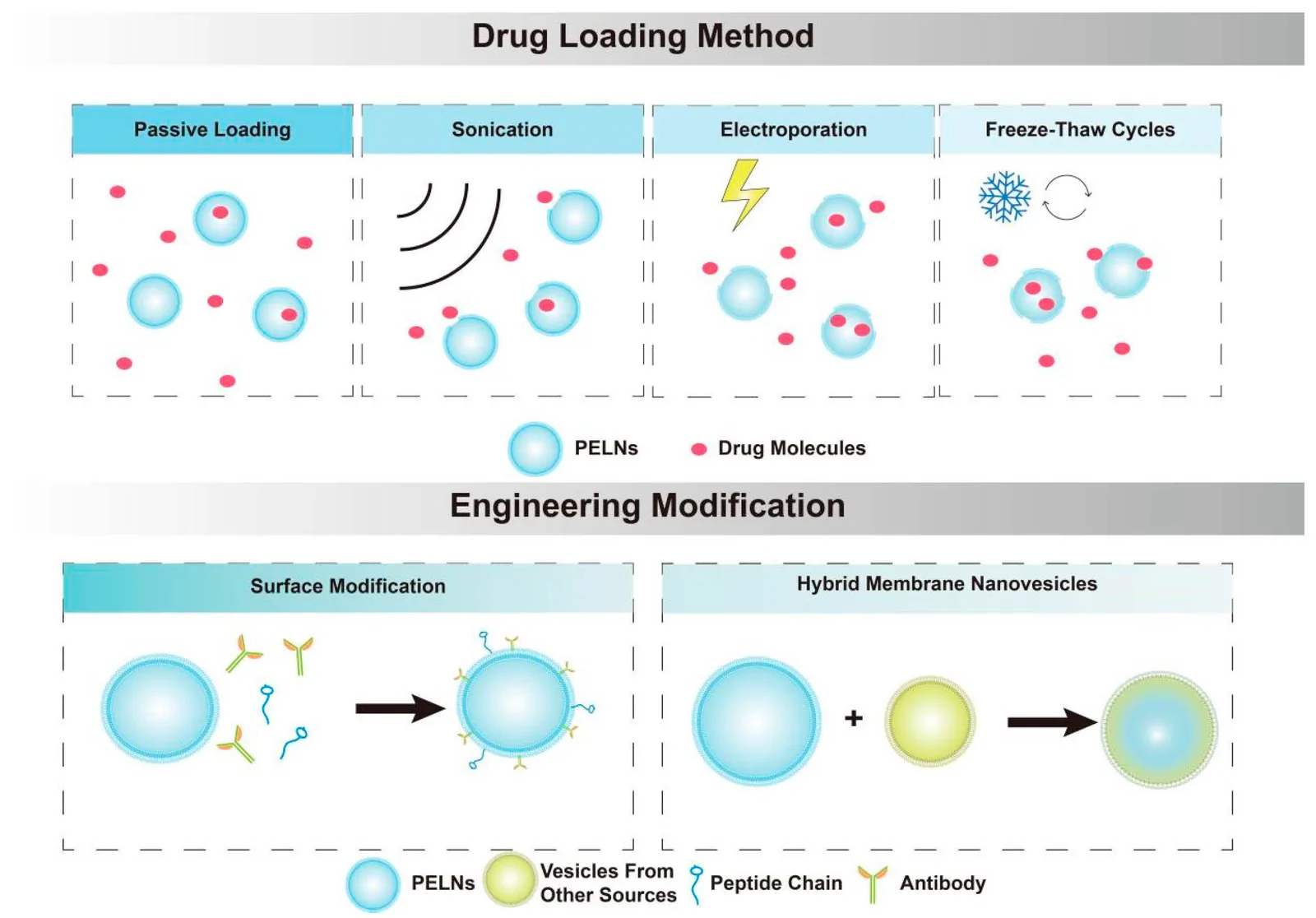
Strategy diagram for constructing PELNs as drug delivery systems. Strategies encapsulate passive diffusion alongside active modalities (sonication and electroporation), complemented by surface engineering platforms (PEGylation, antibody conjugation, and membrane fusion) designed to optimize targeted delivery efficiency and therapeutic efficacy.

Against this background, PELNs, natural nanocarriers, could be used as a novel candidate alternative in next-generation DDSs, whose characteristic shape and composition yield higher biocompatibility and a lower risk of immunogenicity. Moreover, PELNs can efficiently capture various therapeutic molecules (small-molecule drugs, nucleic acids, and proteins) and shield their products from degradation in harsh gastrointestinal environments following oral administration [95]. Compared with traditional synthetic nanoparticles, PELNs can remain drug-stable, avoid enzymatic destruction, and even target tissue more accurately (Figure 4).

#### 4.1.1. Drug Loading Methods

Directly utilizing natural PELNs as drug carriers has the goal of minimizing the introduction of exogenous materials, thereby preserving their inherent biocompatibility and low immunogenic properties. Current loading strategies can be broadly categorized into passive and active methods, each presenting a unique balance between loading efficiency, drug retention, and the preservation of vesicular integrity.

Passive loading (such as co-incubation) uses concentration gradients to permit spontaneous transmembrane passage or insertion into the lipid bilayer. The passive method optimally suits small hydrophobic molecules (e.g., incubating doxorubicin with cabbage PELNs at 37 °C for 4 h to achieve drug loading), while maintaining native vesicle volume [96]. The foremost advantage of passive loading is its non-destructive nature, as it largely preserves the structural integrity, zeta potential, and native surface proteins of PELNs [97]. Drug loading efficiency is low for this method and does not hold for macromolecules (e.g., proteins, nucleic acids) or hydrophilic drugs [98].

To improve loading efficiency and expand the number of available drugs, active loading methods have been developed [91]. Sonication employs high-intensity ultrasound, often in combination with microbubbles or cavitation agents, to transiently generate pores in the vesicular membrane through a phenomenon known as sonoporation. Compared to passive incubation, this method significantly enhances both loading efficiency and post-loading drug retention. For instance, a comparative study evaluating passive incubation, freeze–thaw cycling, and sonication for loading calcitriol into *Tomato*-PELNs revealed that sonication achieved the highest loading efficiency [74]. However, the intense physical shear forces and localized hyperthermia generated during the sonication process pose a severe threat to PELN integrity. These forces can induce extensive vesicle aggregation, degrade sensitive therapeutic payloads, and potentially denature critical surface proteins [97,99].

Electroporation uses high-voltage electrical pulses to create transiently resealing hydrophilic pores. Electroporation effectively overcomes the natural lipid barrier that restricts the delivery of macromolecules. As a notable example, Zhu et al. employed this technique to prepare tumor-exocytosed exosome/AIE luminogen (AIEgen) hybrid nanovesicles, successfully facilitating effective tumor penetration [100]. While the method is effective for loading charged macromolecules (e.g., siRNA, plasmid DNA), it can cause vesicle aggregation and change the distribution of treated samples [101,102]. Furthermore, it should be noted that most successful instances of electroporation currently focus on animal-derived exosomes. Its direct application to PELNs still requires further rigorous experimental validation.

The freeze–thaw cycling method is conducted by repeatedly subjecting a mixture of PELNs and drug solution to rapid freezing in liquid nitrogen and thawing at room temperature. The ice crystals break the ordered structure of the membrane, leading to increased permeability and drug loading [93,101]. For instance, this method was evaluated alongside sonication for loading calcitriol into *Tomato*-PELNs, though it exhibited relatively lower loading efficiency compared to the acoustic cavitation approach [74].

Ultimately, selecting an appropriate drug-loading strategy requires a careful evaluation of the cargo type. While these active physical methods successfully maximize loading efficiency and drug retention, the accompanying mechanical, thermal, or electrical stresses inevitably compromise PELN integrity.

#### 4.1.2. Engineering Modifications

With the help of engineering techniques such as surface modification, membrane fusion, or coating, researchers may significantly enhance the targeting capability and stability of PELNs, thereby making them new drug delivery devices [103]. Some natural PELNs have innate targeting capabilities; for instance, ginger-derived PELNs are preferentially taken up by intestinal stem cells and macrophages to facilitate their target efficacy in inflammatory bowel disease models [31]. Nevertheless, their limited targeting efficiency, specificity, and loading capabilities remain far from the accuracy, flexibility, and multi-purposiveness required for complex diseases.

Covalent surface modification is the main strategy to improve circulatory stability, active targeting, and other biological effects. By coupling peptides, proteins, targets (e.g., folic acid), or chemical compounds (e.g., azide derivatives), the structural integrity of the vesicles can be preserved while endowing them with functionalities such as fluorescence imaging or photothermal/photodynamic effects [103]. Zhanxue Xu et al. [104] covalently conjugated a PD-L1 antibody to licorice PELNs to produce GP@DMX NV. This system not only preserved the intrinsic immunomodulatory nature of PELNs but also, through active targeting, synergistically activated dendritic cells and cytotoxic T lymphocytes in the tumor microenvironment [104]. Su Jin Kang et al. [105] modified the surface of red cabbage-derived PELNs with hyaluronic acid to enable them to target intestinal epithelial cells (via the CD44 receptor) and immune cells. This modification substantially improved their targeted accumulation and therapeutic efficacy in a colitis model [105].

Alternatively, biomimetic hybrid membrane nanovesicles (HMNVs) constructed entirely from biologically derived components can inherit the low immunogenicity and prolonged circulation of PELNs while achieving superior precision targeting [20]. For instance, Wan-Ru Zhuang et al. [106] fabricated bacteria–plant hybrid nanovesicles (BPNs) by fusing *Escherichia coli*-derived outer membrane vesicles (OMVs) with the thylakoid nanovesicles of spinach. These BPNs demonstrated an increased capacity for homing to tumor tissues, promoting immune cell activation and tumor-associated antigen presentation, and eliciting a robust CD8+T lymphocyte response [106]. This approach has opened a novel avenue for the design of multifunctional, synergistic smart nanomedicines.

### 4.2. Fundamental Biological Mechanisms of PELNs

Therapeutically, PELNs utilize their endogenous proteins, lipids, and microRNAs to achieve cross-kingdom regulation in mammalian cells; current studies show that they modulate key biological processes such as anti-tumor, anti-inflammatory, and antioxidant activities or tissue regeneration, making them very promising candidates for clinical translation [107].

#### 4.2.1. Immunomodulation and Anti-Tumor Effects

The uncontrolled inflammatory response is responsible for many pathologies [108]. Therefore, owing to their inherent anti-inflammatory property and good biosafety profile, PELNs represent an extremely promising new intervention method. Studies show that PELNs can achieve anti-inflammatory effects through modulation of immune cell functions and inflammatory cytokine networks [24]. Orally administrated *Purslane*-PELNs successfully alleviate murine DSS-induced colitis via downregulation of pro-inflammatory cytokines and upregulation of the anti-inflammatory cytokine IL-10 in colonic tissue [109]. *Garlic*-PELNs were shown to stably transit the intestinal tract and deliver functional proteins and microRNAs conferring protection against ulcerative colitis (UC) by Xiaoyuan Wang [110]. Additionally, *Houttuynia cordata* [111], ginger [112], and ginseng [113,114] have all been proven to have a significant therapeutic effect on gastrointestinal inflammation. Moreover, the anti-inflammatory effects of PELNs do not only exist within the digestive tract. *Rehmannia glutinosa*-PELNs have favorable therapeutic efficacy towards pulmonary inflammation [115]. Meng Long et al. found that *Dandelion*-PELNs could exert anti-inflammatory effects by regulating macrophage M1/M2 polarization, thus ameliorating symptoms of allergic dermatitis [116]. In a model of ultraviolet-induced skin photoaging and collagen degradation, lavender-derived PELNs greatly inhibit cutaneous inflammation, inflammatory cell infiltration, and collagen loss and restore normal hair follicle arrangement [117].

Crucially, this robust capacity for immune microenvironment regulation, particularly macrophage reprogramming, directly underpins their anti-tumor mechanisms. For immunomodulation, *Artemisia annua*-PELNs transport endogenous plant mitochondrial DNA (mtDNA) into tumor-associated macrophages (TAMs), thereby activating the cGAS-STING signaling pathway and reprogramming pro-tumorigenic M2-type TAMs into an anti-tumor M1 phenotype [118]. Likewise, Jeong Moo Han et al. showed that *Eutrema japonicum*-PELNs also show immunostimulatory and tumor-suppressive capabilities [119]. Concurrently, PELNs trigger direct cytotoxic effects on malignancies. *Lemon*-PELNs specifically trigger activation of the TRAIL/DR5 apoptotic pathway for inhibiting tumor proliferation with no significant toxicity towards normal cells [120]. In addition, many other plant-derived PELNs like aloe [121], grape [50], and garlic [122] have been shown to demonstrate anti-tumor effects in preclinical studies; the underlying mechanisms involve several aspects, e.g., cell cycle arrest, apoptosis induction, and regulation of immune cell function.

#### 4.2.2. Antioxidant and Tissue Repair Functions

Abnormally elevated levels of reactive oxygen species (ROS), e.g., O_2_^−^, H_2_O_2_, and •OH, can induce severe oxidative damage to biological macromolecules, leading to cellular dysfunction or apoptosis [123]. Although various substances, such as vitamin C, vitamin E, and coenzyme Q10, have demonstrated antioxidant activity, their clinical efficacy is often limited by factors including low bioavailability and poor stability.

Numerous studies have confirmed that PELNs can alleviate oxidative damage by directly scavenging ROS, upregulating the expression of endogenous antioxidant enzymes (e.g., superoxide dismutase [SOD] and catalase [CAT]), and activating key antioxidant signaling pathways such as the Kelch-like ECH-associated protein 1 (Keap1)–nuclear factor erythroid 2-related factor 2 (Nrf2) pathway [124]. Jie Cao et al. reported that red cabbage-derived PELNs reduce intracellular ROS levels and attenuate mitochondrial dysfunction [125]. Similarly, *Ginseng*-PELNs protect skin cells from ultraviolet radiation- and oxidative stress-induced apoptosis by inhibiting the activator protein-1 (AP-1) pathway [45]. Zixuan Sun et al. demonstrated that aloe-derived PELNs possess the ability to scavenge ROS generated by UVA and UVB irradiation, thereby protecting the skin from photodamage [126]. Furthermore, Ting Wang et al. reported that coriander-derived PELNs are effectively internalized by HaCaT cells and mouse skin tissue. These nanoparticles not only promote cell migration but also enhance ROS clearance by upregulating antioxidant enzyme expression [127].

This robust ability to regulate the cellular oxidative microenvironment is highly conducive to tissue regeneration, especially for chronic non-healing diabetic wounds exacerbated by persistent inflammation and hyperglycemic injury. It has been shown that *Lemon*-PELNs facilitate diabetic wound healing through modulation of macrophage polarization as well as promotion of proliferation and migration of vascular endothelial cells and fibroblasts [128]. In contrast, ginseng-derived PELNs enhance endothelial function during hyperglycemia via modulation of cellular metabolic reprogramming (upregulation of glycolysis but suppression of aberrant oxidative phosphorylation), thereby driving angiogenesis and providing nutrition for ulcer repair [129].

PELNs have also demonstrated efficacy in other areas. For instance, *Gouqi*-PELNs are internalized by mouse embryonic osteoblast MC3T3-E1 cells in vitro and promote their proliferation and differentiation. In a mouse fracture model, treatment with *Gouqi*-PELNs resulted in increased trabecular thickness and bone marrow density, suggesting that these nanoparticles facilitate fracture healing [130].

#### 4.2.3. Other Therapeutic Activities

In addition, the therapeutic potential of PELNs extends to other applications including cardiovascular protection and metabolic homeostasis regulation. Tomato-derived PELNs activate the Keap1-Nrf2 antioxidant pathway via delivery of miR-164a/b-5p, inhibiting platelet-derived growth factor-BB (PDGF-BB)-induced abnormal proliferation and migration of vascular smooth muscle cells, thus suggesting their potential use in the prevention of vascular remodeling diseases like atherosclerosis [131]. An effect of PELNs on the gut microbiota has been found, too. Citrus-derived PELNs modulate the gut microbiota composition in a high-fat diet model, decreasing the abundance of pathogenic bacteria (e.g., *Prevotellamassilia timonensis*) and enhancing the abundance of beneficial bacteria (e.g., *Acetatifactor muris*); therefore, they improve the metabolic environment [132]. Additionally, Cathrine M. S. Bethi et al. discovered that fennel-derived PELNs have abundant phytoferritin content that could be absorbed using various gastrointestinal uptake mechanisms, possibly providing a new therapeutic option for iron deficiency anemia [133]. However, the exact intracellular mechanism involved in the release of ferritin from these nanoparticles into the systemic circulation still needs further investigation.

## 5. Therapeutic Potential of PELNs in Common Liver Diseases

**Figure 5:**
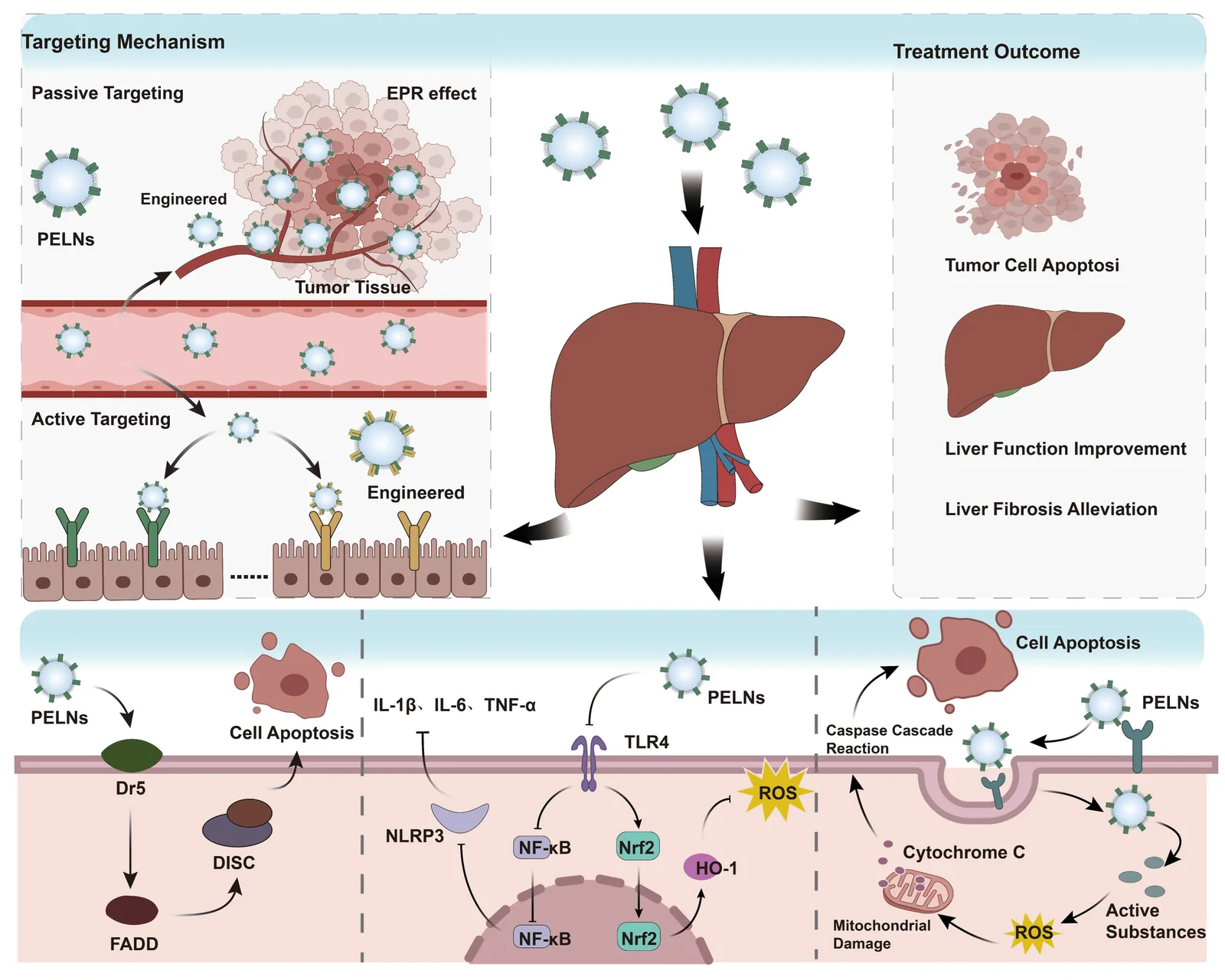
Schematic diagram illustrating the multi-target mechanisms of PELNs in liver disease therapy. This figure comprehensively depicts liver targeting via receptor-mediated pathways or the EPR effect, as well as hepatoprotective effects achieved by inhibiting the TLR4/NF-κB/NLRP3 inflammatory axis, activating the Nrf2 antioxidant pathway, and regulating apoptosis and ferroptosis.

The therapeutic potential of PELNs is well established in various disease models such as malignancies and metabolic disorders because they possess intrinsic biological activities (e.g., anti-inflammatory and regenerative effects) along with excellent biocompatibility, low immunogenicity, and high stability. As an organ responsible for performing essential functions like metabolism, detoxification, and biosynthesis, the liver requires effective therapies balancing efficacy and safety. These characteristics make PELNs a very promising novel nanoplatform in the field of liver disease therapy. Research on this topic has been rapidly increasing. In this section, we review the therapeutic applications and potential molecular mechanisms of PELNs in major liver disease models, aiming to offer a theoretical foundation for developing new innovative PELN-based therapeutic strategies for liver diseases (Figure 5).

### 5.1. Applications in Common Liver Diseases

Animal-derived exosomes’ therapeutic efficacy has set up an important paradigm in liver disease intervention. For instance, Seohyun Kim et al. developed high-purity/high-yield EVs derived from engineered human mesenchymal stem cells expressing SIRPα (natural ligand of CD47) on their surface, which were used for treating acute liver injury, showing favorable scalability and translational potential [134]. Another study demonstrated that MSC-Ex could effectively alleviate symptoms of HF induced by DEN/CCl_4_ in rats [135]. Yan Lin et al. proposed a new strategy using MSC-Ex modified with activated hepatic stellate cell (aHSC)-targeting peptides for the treatment of liver fibrosis [136]. While these studies demonstrate the tremendous therapeutic efficacy of animal-derived exosomes, issues such as large-scale production, high cost, and possible immunogenicity have limited their clinical translation. Overcoming these translational bottlenecks, PELNs provide a promising alternative for liver disease therapy. Besides being scalable and safe natural carriers, they also exhibit significant intrinsic therapeutic bioactivity, providing a powerful dual-functional strategy in various liver disease models.

#### 5.1.1. Hepatocellular Carcinoma

HCC is responsible for about 90% of all liver cancer cases [137] and has become one of the focuses of current PELN research. Tahereh Tajik et al. studied *Cannabis*-PELNs obtained from two chemotypes, Ark-01 (CBD-dominant) and Krmn-01 (THC-dominant), and explored their anti-hepatoma potential [138]. The study confirmed that *Cannabis*-PELNs especially enriched with CBD act as natural carriers for bioactive phytochemicals, inhibiting hepatoma cell growth through induction of cell cycle arrest and mitochondria-mediated apoptosis. These in vitro results need additional in vivo validation on their specific active components, therapeutic efficacy, and safety profile. Gao et al. reported that *Black Mulberry*-PELNs have anti-tumor effects both in vitro and in an orthotopic mouse model of HCC [139]. *Black Mulberry*-PELNs are rich in bioactive glycolipids and flavonoids and target hepatoma cells through galactose receptor-mediated endocytosis. Inside the cells, they increase the levels of ROS, causing mitochondrial dysfunction, thus inhibiting cell proliferation and migration and inducing apoptosis. In vivo, *Black Mulberry*-PELNs are stable inside the gastrointestinal tract, accumulate in liver tumors through enterohepatic circulation, and modulate gut microbiota balance, effectively suppressing tumor growth and improving liver function parameters. Moreover, Zhang et al. isolated *Asparagus*-PELNs and examined their anti-proliferative activity towards HCC and their pro-apoptotic mechanisms. Also, PEGylation was used to improve the anti-clearance capability and tumor-targeting capacity of *Asparagus*-PELNs [140]. In vitro, *Asparagus*-PELNs enter cancer cells through phagocytosis, upregulate the expression of BAX, BAK, and AIF, activate Caspase-9, and cleave PARP, resulting in tumor cell apoptosis. To overcome the rapid clearance by the mononuclear phagocyte system in vivo, the researchers modified *Asparagus*-PELNs with PEG. The modification greatly increased their blood circulation time. Together with the enhanced permeability and retention (EPR) effect inherent to solid tumors, this resulted in an increase in their accumulation within tumor tissue.

#### 5.1.2. Hepatic Fibrosis

Beyond HCC, HF constitutes another major focus of PELN research. Although the liver possesses remarkable regenerative capacity, recurrent and persistent injury can drive HF, a chronic disorder characterized by an excessive wound-healing response and substantial extracellular matrix (ECM) accumulation [141]. HF represents a common endpoint for various chronic liver diseases, including MAFLD and viral hepatitis, and constitutes a high-risk pathological basis for the development of HCC. In a study focusing on *Hemp*-PELNs, Kim et al. demonstrated through in vivo experiments that *Hemp*-PELNs inhibited oxidative stress and reduced fibrotic marker proteins, exhibiting hepatoprotective effects against WD/FFC-induced HF [142]. Another study indicated that *Ginger*-PELNs targeted and ameliorated mitochondrial dysfunction, effectively inhibiting the activation of HSCs and subsequently delaying or preventing the progression of HF [143].

MAFLD, formerly known as non-alcoholic fatty liver disease (NAFLD), is currently the most common etiology of HF worldwide [144]. Both cellular and in vivo experiments have demonstrated that *blueberry*-PELNs ameliorate MAFLD by effectively attenuating mitochondrial oxidative stress [145]. In a murine model of liver disease, *Artemisia capillaris*-PELNs were shown to reduce lipid deposition, improve liver function, and attenuate the progression of HF [146]. Notably, the researchers identified 293 microRNAs (miRNAs) in *Artemisia capillaris*-PELNs. Compared with the miRNAs present in *Arabidopsis thaliana*-PELNs, gma-miR5368, which plays a critical role in the antioxidant and anti-pathogen defense mechanisms of plants, exhibited the highest abundance. Although differential targeting analyses indicate that these miRNAs participate in essential cellular processes and lipid metabolism, their specific therapeutic mechanisms in MAFLD remain to be further elucidated. Furthermore, PELNs derived from sources such as tangerine peel [147], garlic [148,149], and lemon [150] have also demonstrated therapeutic efficacy against HF and MAFLD.

#### 5.1.3. Alcohol-Related Liver Disease

Alcohol is a common injurious factor to the liver. ALD constitutes a major cause of liver-related morbidity and mortality worldwide. Its disease spectrum ranges from simple steatosis to alcoholic steatohepatitis (ASH) and can further progress to severe alcoholic hepatitis (AH), HF, and even liver failure. Studies indicate that PELNs demonstrate potential in intervening in ALD.

*Pomegranate*-PELNs have shown significant efficacy in preventing binge alcohol-induced liver injury in a mouse model. Compared to the model group, mice preconditioned with *Pomegranate*-PELNs exhibited significantly reduced levels of oxidative stress markers (e.g., CYP2E1, 3-nitrotyrosine) and apoptosis-related proteins in both the liver and intestine following repeated alcohol gavage (6 g/kg) [151]. *Pueraria lobata*-PELNs exert their hepatoprotective effects by modulating key enzymes involved in alcohol metabolism. One study reported that pre-treatment with *Pueraria lobata*-PELNs significantly enhances hepatic aldehyde dehydrogenase (ALDH) activity while concurrently inhibiting alcohol dehydrogenase (ADH). This dual modulation accelerates the clearance of the toxic metabolite acetaldehyde and potentially reduces its generation, leading to concomitant decreases in ethanol and acetaldehyde levels in both the liver and serum. More importantly, this study further revealed that *Pueraria lobata*-PELNs suppress the expression of key proteins associated with ferroptosis, indicating that they alleviate ALD through a dual pathway: promoting alcohol metabolism and inhibiting programmed cell death [152]. Furthermore, ginger-derived PELNs have also been confirmed to possess activity against alcoholic liver injury, with their effects likely attributable to the inherent potent antioxidant and anti-inflammatory components of ginger [153].

Taken together, these studies have shown that although PELNs derived from various plants have unique molecular targets (modulating metabolic enzymes or inhibiting ferroptosis), there are common protective mechanisms such as mitigation of oxidative stress, suppression of inflammatory responses, and regulation of cell death shared by them all.

#### 5.1.4. Acute Liver Failure

ALF is an extremely serious clinical condition arising from different etiologies (e.g., hepatotropic virus infection; drug or toxin poisoning), characterized by massive hepatocyte necrosis with acute hepatic decompensation over a short time frame, leading to high mortality rates, with few available treatments clinically [154]. Over the last several years, many preclinical studies, mainly based on mouse models, have demonstrated the promising therapeutic potential of PELNs in ALF. They revealed profound hepatoprotection via diverse molecular mechanisms.

For example, *Honeysuckle*-PELNs can relieve ALF through modulation of the “gut–liver axis”, which might be achieved by preserving intestinal barrier function to mitigate systemic inflammation [155]. Both oral and intravenous administration of *Honeysuckle*-PELNs significantly alleviated hepatocyte necrosis and inflammatory infiltration in a lipopolysaccharide (LPS)/D-galactosamine (D-GalN)-induced ALF mouse model. They markedly decreased serum alanine aminotransferase and aspartate aminotransferase (approximately 53% decrease compared to the model group, *p* < 0.01) and increased the survival rate. Consistent with these findings, histopathological analysis of liver tissue showed significant attenuation of hepatic injury. Similarly, *Garlic*-PELNs showed multifaceted hepatoprotection for LPS/D-GalN-induced injury by suppressing inflammatory responses (possibly via inhibition of the NLRP3 inflammasome), regulating apoptosis and pyroptosis, and reducing pathological infiltration of immune cells into the liver [149]. *Grape*-PELNs also displayed similar hepatoprotective effects to those in LPS/D-GalN-induced ALF. They significantly lowered serum alanine aminotransferase and aspartate aminotransferase activities and simultaneously inhibited the release of key pro-inflammatory cytokines such as interleukin-6 (IL-6), interleukin-1β (IL-1β), and tumor necrosis factor-α (TNF-α). Thus, *Grape*-PELNs possess great translational potential as a novel clinical therapeutic strategy [156]. Zheng et al. found that *Artemisia argyi*-PELNs could significantly ameliorate hepatic pathological damage and lower systemic inflammatory cytokine levels in LPS/D-GalN-induced ALF mice [157].

### 5.2. Therapeutic Mechanisms in Liver Disease

Thoroughly understanding the mechanisms of PELNs in treating liver diseases can help us optimize their targeted delivery efficiency, rationally design PELN-based combination therapeutic strategies, and develop next-generation intelligent nanomedicines. Here, we focus on three crucial mechanisms of how PELNs exert hepatoprotection, including (1) targeting mechanisms leading to liver-specific enrichment; (2) anti-inflammatory effects via modulating key inflammatory pathways; and (3) antioxidant effects by activating endogenous defense systems.

#### 5.2.1. Hepatocyte Targeting Mechanisms

Achieving liver-specific delivery is a prerequisite for the therapeutic efficacy of PELNs. Studies have demonstrated that various PELNs exhibit a natural propensity to accumulate in liver tissues in vivo. This tropism is primarily mediated by two complementary mechanisms: active targeting and passive targeting.

Active targeting refers to the specific binding of molecules on the PELN surface to cognate receptors on target cells within the liver tissue, thereby mediating vesicle recognition, adhesion, and endocytosis. Although the tissue tropism of animal-derived exosomes is closely associated with surface proteins such as integrins and tetraspanins (e.g., CD63 and CD81), direct investigations into analogous targeting proteins on PELNs remain limited. Nevertheless, existing evidence suggests that similar targeting proteins may be present on the surface of PELNs. One study reported that *Garlic*-PELNs achieve efficient cellular uptake by binding to CD98 heavy chain receptors, which are highly expressed on the surface of hepatoma cells [158]. When the surface proteins of *Garlic*-PELNs are removed by protease treatment, or when CD98 receptors on hepatoma cells are blocked with antibodies, their uptake efficiency is significantly reduced. This finding implies that PELNs can target liver cells through a “protein–receptor” interaction mechanism. Furthermore, certain surface proteins in liver tissue possess the ability to specifically recognize particular glycan chains. For example, the asialoglycoprotein receptor (ASGPR) can specifically recognize and bind glycoproteins with exposed terminal galactose or N-acetylgalactosamine residues [159]. If PELNs display similar glycan chain structures on their surface, then they could be recognized by ASGPR, thereby achieving active targeting.

Passive targeting takes advantage of the physiological or pathological features of the liver itself to physically trap PELNs, resulting in their accumulation inside the liver. In pathological situations like HCC or liver fibrosis, the permeability of local neovascularization in the liver increases while lymphatic drainage is impeded. This allows PELNs to extravasate out of blood vessels and be retained in diseased tissues, which is called the EPR effect [160]. Interestingly, the EPR effect is one major way nanomedicines realize passive targeting, especially in tumors or inflamed sites. For example, the small-molecule nanomedicine engineered by Li et al. was able to target liver tissue through the EPR effect [161]. Similarly, the drug-loaded nanovesicles engineered by Zheng et al. took advantage of this effect to dramatically increase targeted drug retention at the tumor site [160]. Exploiting the EPR effect relies heavily on nanoparticle size (usually 10–200 nm) and surface charge. So far, no study has demonstrated systematically that natural PELNs take advantage of this mechanism for hepatic accumulation due to the unoptimized physicochemical properties of these particles for effective EPR-mediated extravasation. This provides motivation for engineering efforts, including modulating particle size, tuning surface charge, or introducing PEGylation, which will greatly improve pharmacokinetic profiles. As mentioned above, the PEGylated *Asparagus*-PELNs have shown promising results in effectively exploiting the EPR effect for liver-specific delivery. A surface engineering approach could equip PELNs with efficient EPR-driven hepatic drug delivery.

#### 5.2.2. Anti-Inflammatory Effects

Chronic inflammation is an important pathological process that drives almost any type of liver disease. Natural carriers of bioactive compounds, PELNs are able to efficiently suppress inflammatory responses through targeting key inflammatory pathways including NF-κB and NLRP3.

*Artemisia argyi*-PELNs have profound anti-inflammatory and hepatoprotective effects on ALF. The mechanisms are related to the synergistic regulation of inflammation and oxidative stress [157]. *Artemisia argyi*-PELNs can inhibit the TLR4/NF-κB/NLRP3 axis to alleviate inflammation and apoptosis and meanwhile activate Nrf2-CYP2A5 to alleviate oxidative stress. Besides these, they also downregulate the CCL5/7-CCR5 and CXCL9/10/11-CXCR3 chemoattractant axes, thereby reducing hepatic immune cell infiltration and reshaping the immune microenvironment. Integrative omics found MIR2916, which is greatly enriched in *Artemisia argyi*-PELNs, as an important functional regulator for this TLR4 signaling cascade.

*Garlic*-PELNs exhibit strong anti-inflammatory and anti-apoptotic effects against ALF models [149]. The wide-ranging mechanisms of action are as follows: inhibiting the NF-κB signaling pathway by reducing serum pro-inflammatory cytokines (IL-6, IL-1β, and TNF-α), modulating the apoptosis-related proteins (reducing the Bax/Bcl-2 ratio and inhibiting Caspase-9/3 cleavage), and suppressing NLRP3 inflammasome assembly and activation to reduce Caspase-1 and IL-18 maturation at once. In line with earlier work on other compounds such as curcumin, which attenuates liver injury via inhibition of NF-κB [162,163,164,165], the literature reviewed here supports that PELNs regulate inflammatory pathways through multiple targets.

In addition, lessons learned from PELN research on other systemic inflammatory conditions cross-pollinate the liver field. Mulberry bark-derived ELNs promote mucosal barrier function via the activation of the AhR signaling pathway [166]. Because of the crucial role of AhR signaling in hepatic immune regulation and inflammation control [167], it is exciting to consider how PELNs might modulate the liver microenvironment via similar pathways as a frontier area for future research.

#### 5.2.3. Hepatic Antioxidant Mechanisms

Building upon the previously described general antioxidant properties of PELNs, combating oxidative stress is particularly paramount in hepatology, as it is deeply involved in the pathogenesis of ALD, MAFLD, HF, and HCC. The bioactive compound shogaol from *Ginger*-PELNs activates the transcription factor Nrf2 via the TLR4/TRIF pathway, leading to its nuclear translocation, where it induces the expression of several antioxidant genes such as heme oxygenase-1 (HO-1), thus suppressing ROS production at their source [153]. It has also been found that BELNs exhibit hepatoprotection through the activation of the Nrf2 pathway [145]. Activating the Nrf2 pathway with *Blueberry*-PELNs could sustainably increase hepatocytes’ resistance against oxidative damage. Such an approach would be more efficient than directly scavenging ROS and would provide long-term protection.

The current research regarding the antioxidant effect of PELNs in liver diseases is still at an early stage. Thus, most PELNs’ molecular mechanisms and antioxidant activities are unknown. Further work will be focused on screening PELNs derived from various plant sources that possess potent antioxidant potential. In addition to the Nrf2 pathway, it is important to explore whether there exist other mechanisms by which PELNs exert their antioxidant effects (e.g., via a Keap1-independent mechanism).

Table 3 presents the treatment of liver diseases by various PELNs in more detail.

## 6. Conclusions and Future Perspectives

In the last two decades, PELNs have shown tremendous potential as a novel biomedical platform, especially in liver diseases, due to their wide availability, simplicity of preparation, structural stability, and dual abilities: inherent biological activities and effective drug delivery. Currently, their therapeutic application in hepatology relies almost entirely on preclinical in vivo evaluations. As detailed in Section 5.1, researchers frequently employ specific rodent models (including CCl4-induced liver fibrosis, HFD-induced NAFLD, and LPS/D-GalN-induced acute liver failure) to confirm that PELNs can effectively alleviate hepatic injury. Regrettably, no clinical trials of PELNs for liver diseases have been conducted to date. However, the translation from promising preclinical candidates to reliable clinical therapeutics has faced some major hurdles. Encouragingly, the first steps towards clinical exploration have been initiated for PELNs in several non-hepatic indications, including oral mucositis (NCT01668849), inflammatory bowel disease (NCT04879810), and colorectal delivery of curcumin (NCT01294072), although one trial (NCT03493984) was withdrawn without enrollment [124].

Despite these promising candidates, translating PELNs into reliable clinical therapeutics faces major hurdles rooted in standardization and safety. Upstream, plants vary inherently by species, cultivar, growing environment, and harvest time. This inherent variability can substantially influence downstream preclinical and clinical outcomes. At the biophysical level, variations in lipid composition can alter vesicular membrane fluidity and surface properties, which may affect drug loading capacity and receptor-mediated targeting, potentially leading to variable in vivo pharmacokinetics. In terms of encapsulated payloads such as metabolites and miRNAs, environmental fluctuations may cause batch-to-batch variation in biological activity, making consistent therapeutic efficacy difficult to maintain. Downstream, this physicochemical heterogeneity poses a major challenge to reproducibility. Seasonal changes in plant tissues can reduce the efficiency of standard extraction procedures, making batch-to-batch consistency for Good Manufacturing Practice (GMP) compliance difficult. The variable presence of uncharacterized plant proteins or antigens raises potential concerns about immunogenicity, although current evidence suggests that plant-derived vesicles are generally poorly immunogenic; this uncertainty further complicates the regulatory classification of PELNs.

Beyond inherent plant variability, the absence of unified preparation protocols constitutes another major barrier. Currently, products obtained using different methods (such as differential centrifugation, SEC, and polymer precipitation) exhibit severe discrepancies in purity and vesicle integrity. Addressing these intertwined challenges necessitates integrating controlled agricultural systems to secure a uniform baseline biomass with optimized downstream processing. Ultimately, this integration is the fundamental prerequisite for developing a GMP-compliant manufacturing framework. Finally, comprehensive toxicological assessments and clear regulatory classifications regarding whether naturally derived versus engineered vesicles require different evaluation frameworks must be established to guarantee clinical safety and accelerate translation.

**Figure 6:**
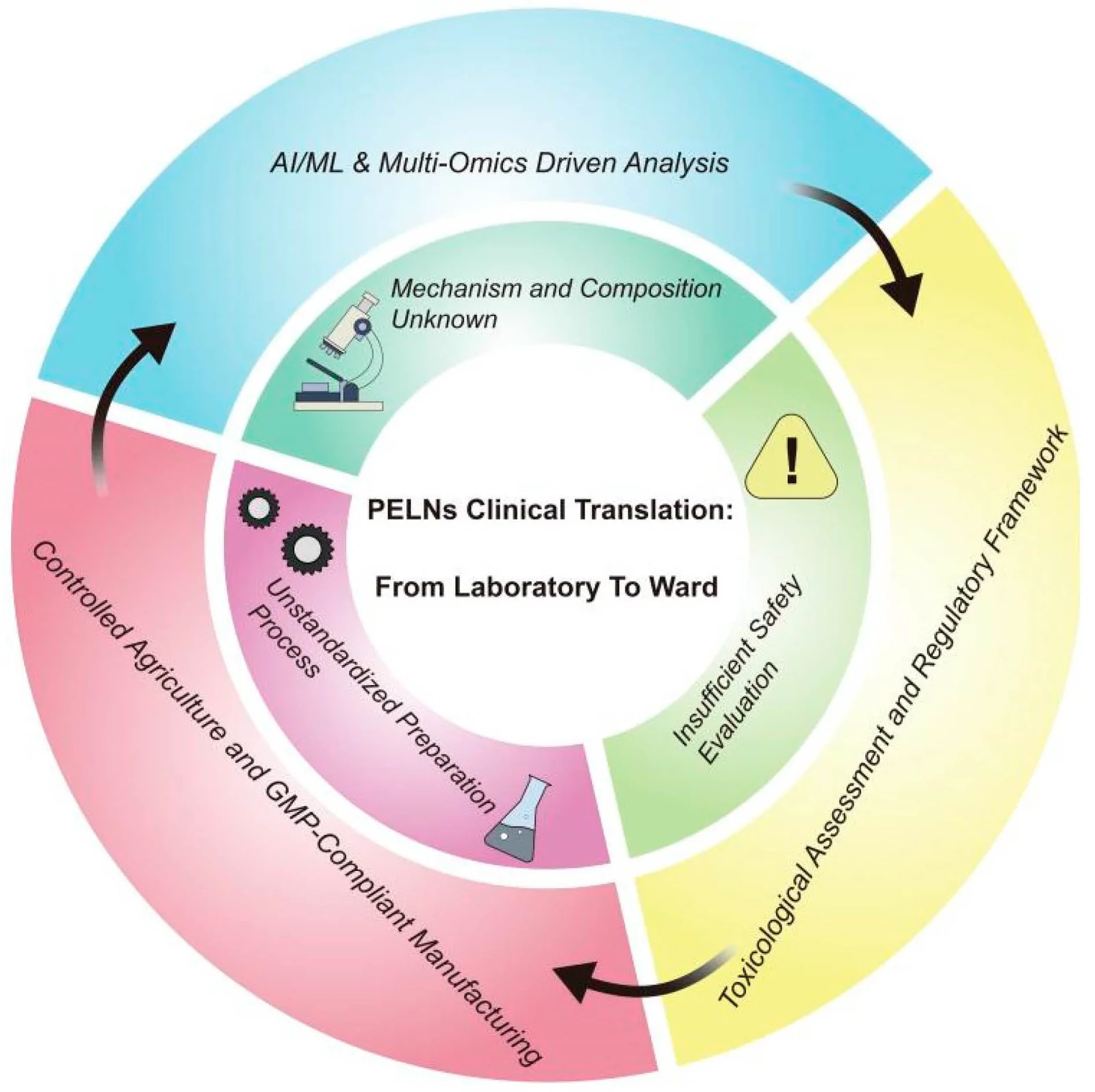
Clinical translation of PELNs in liver diseases: challenges and countermeasures.

To address the aforementioned bottlenecks in mechanistic elucidation and scalable preparation, interdisciplinary integration provides critical new tools. Specifically, artificial intelligence (AI) and machine learning (ML) are expected to significantly advance PELN research paradigms. By integrating multi-omics data (proteomics, lipidomics, small RNAomics), ML algorithms can mine hidden associations between complex PELN components and specific biological effects, thereby accelerating mechanistic elucidation. Concurrently, AI-driven process optimization can screen optimal extraction parameters, achieving intelligent trade-offs among purity, yield, and cost to facilitate standardized, scalable production. Furthermore, advancements in nanotechnology and synthetic biology will open precise pathways for the engineering modification of PELNs (e.g., targeting modification, intelligent drug loading) (Figure 6).

## Figures and Tables

**Table 1:** Plant-derived small-molecule compounds and their functions.

Small Molecule Category	Specific Compound	Source Plant	Function/Activity	Reference
Curcuminoids	Curcumin	Turmeric	Anti-Inflammatory, Antioxidant, Anti-Tumor	[38,39]
Flavonoids	Rutin, Naringin, Hesperidin	Citrus (Lemon, Grapefruit, Orange)	Antioxidant, Anti-Inflammatory, Cardiovascular Protection	[41,42,43,44]
Carotenoids	β-Carotene	Carrot	Antioxidant, Vitamin A Precursor	[40]
Saponins	Ginsenoside	Ginseng	Immunomodulation, Anti-Fatigue, Neuroprotection	[45,46,47]
Phenolic Compounds	Gallic Acid, Ellagic Acid, Catechin	Grape, Blueberry, Tea	Strong Antioxidant, Anti-Inflammatory	[48,49,50,51]
Organic Acids	Citric Acid	Lemon	Antioxidant, Immunomodulation	[52]
Glucosinolates/Isothiocyanates	Sulforaphane, Allyl Isothiocyanate	Broccoli	Anti-Cancer (Activates Nrf2 Pathway)	[53,54,55]

**Table 2:** Comparison of PELN extraction/purification techniques.

Isolation Method	Separation Principle	Yield	Purity	Scalability	Drawbacks
dUC	Size & sedimentation velocity (>100,000 g)	Moderate to High	Low to Moderate	Poor	Co-sedimentation of protein aggregates; high shear stress risks membrane damage
DGUC	Buoyant density (1.10–1.19 g/mL)	Low	Very High	Very Poor	Highly labor-intensive (12–18 h); hyperosmotic media may induce vesicle shrinkage
UF	Molecular weight cutoff exclusion	Moderate to High	Low to Moderate	High	Membrane fouling and co-retention; minor hydrostatic shear, but native bioactivity retained
SEC	Hydrodynamic radius/molecular exclusion	Moderate	High	Moderate	Limited sample volume; low-shear elution perfectly preserves vesicle morphology and Zeta potential
PEG	Polymer-driven hydration depletion	High	Low	High	Co-precipitates non-specific macromolecules; residual PEG alters Zeta potential and masks surface ligands
IAC	Specific antigen-ligand/antibody capture	Very Low	Very High	Very Poor	Lack of universal plant markers; harsh elution conditions risk denaturing surface proteins

**Table 3:** Summary of therapeutic applications and mechanisms of PELNs in liver diseases.

Disease Type	Plant Source	Therapeutic Mechanisms and Biological Effects	Key Molecules/Pathways
HCC	Cannabis	Inhibiting the viability of hepatoma cells, arresting the cell cycle at the G0/G1 phase, and inducing mitochondria-dependent apoptosis, with no significant effects on normal cells	Bcl-2/BAX, CASP3/CASP8/CASP9, G0/G1 Phase Arrest
	Black Mulberry Leaf	This process is driven by galactose receptor-mediated endocytosis, which elevates intracellular ROS to induce mitochondrial dysfunction, and also modulates gut microbiota balance	Galactose Receptor, ROS
	*Asparagus cochinchinensis*	This process upregulates BAX, BAK, and AIF, triggers Caspase-9/PARP apoptosis, and employs PEGylation to improve circulation and the EPR effect	BAX, BAK, AIF, Caspase-9, PARP
HF/MAFLD	Hemp	inhibit Oxidative Stress; Reduce Fibrosis Marker Proteins	/
	Ginger	Promoting fusion and inhibiting fission restored mitochondrial dynamics, thereby ameliorating dysfunction, protecting hepatic stellate cells, and attenuating fibrosis	Mitochondrial Fusion/Fission (Mfn/DRP1)
	Blueberry	Mitochondrial oxidative stress was alleviated, and the Nrf2 pathway was activated	Nrf2 pathway
	*Artemisia capillaris*	Lipid deposition was reduced, and liver function was improved. The involvement of gma-miR5368 in lipid metabolism was identified	gma-miR5368
	Lemon	ROS levels were decreased, antioxidant signaling was upregulated, and glycolipid metabolism and systemic redox homeostasis were enhanced	Nrf2/HO-1
	Tangerine peel	Improving glucose and lipid metabolism, reducing hepatic lipid accumulation, restoring the gut microbiota, activating fatty acid β-oxidation and glycolysis	AMPK/SREBP-1, UCP1/PPAR-α, GLUT2/PEPCK
ALD	Pomegranate	Reducing the levels of oxidative stress markers (e.g., CYP2E1, 3-nitrotyrosine) and apoptosis-related proteins in the liver and intestine	CYP2E1, 3-nitrotyrosine
	*Pueraria lobata*	Enhancement of aldehyde dehydrogenase (ALDH) activity; inhibition of alcohol dehydrogenase (ADH) activity; suppression of ferroptosis	ALDH, ADH, ferroptosis-related proteins
	Ginger	The preventive and therapeutic effects against alcoholic liver injury are attributed to its potent antioxidant and anti-inflammatory components	TLR4/TRIF pathway, Nrf2, HO-1
ALF	Honeysuckle	Regulates the “gut–liver axis”; reduces serum ALT and AST levels; inhibits the release of inflammatory cytokines	IL, IL-1β, TNF-α
	*Allium sativum* (Garlic)	Suppresses inflammatory responses; regulates apoptosis and pyroptosis; reduces pathological infiltration of immune cells in the liver	NF-κB, NLRP3, Bax/Bcl-2, Caspase-1
	*Vitis vinifera* (Grape)	Decreases ALT and AST activities; blocks the release of inflammatory cytokines	IL6, IL-1β, TNF-α
	*Artemisia argyi*	Alleviates inflammation and apoptosis by inhibiting the TLR4/NF-κB/NLRP3 axis, and reduces oxidative stress via Nrf2-CYP2A5 activation	TLR4/NF-κB/NLRP3 axis, Nrf2-CYP2A5

## Data Availability

No new data were created or analyzed in this study. Data sharing is not applicable to this article.

## Abbreviations

The following abbreviations are used in this manuscript:

ABC	ATP-binding cassette
ADH	Alcohol dehydrogenase
AH	Alcoholic hepatitis
ALD	Alcohol-related liver disease
ALDH	Aldehyde dehydrogenase
ALF	Acute liver failure
AP-1	Activator protein-1
ASGPR	Asialoglycoprotein receptor
ASH	Alcoholic steatohepatitis
BPNs	Bacteria–plant hybrid nanovesicles
CAT	Catalase
Cryo-EM	Cryo-electron microscopy
DDS	Drug delivery systems
DGDG	Digalactosyldiacylglycerol
DGUC	Density gradient ultracentrifugation
DLS	Dynamic light scattering
DTT	Dithiothreitol
dUC	Differential ultracentrifugation
ECM	Extracellular matrix
EPR	Enhanced permeability and retention
ESCRT	Endosomal sorting complex required for transport
EVs	Extracellular vesicles
EXPO	Exocyst-positive organelle
D-GalN	D-galactosamine
GMP	Good Manufacturing Practice
GPX1	Glutathione peroxidase 1
HCC	Hepatocellular carcinoma
HF	Hepatic fibrosis
HMNVs	Hybrid membrane nanovesicles
HO-1	Heme oxygenase-1
HSCs	Hepatic stellate cells
IAC	Immunoaffinity capture
IBD	Inflammatory bowel disease
ICIs	Immune checkpoint inhibitors
IL-1β	Interleukin-1β
IL-6	Interleukin-6
ILVs	Intraluminal vesicles
IVVs	Intravacuolar vesicles
Keap1	Kelch-like ECH-associated protein 1
LPS	Lipopolysaccharide
MAFLD	Metabolic dysfunction-associated fatty liver disease
MGDG	Monogalactosyldiacylglycerol
MISEV	Minimal Information for Studies of Extracellular Vesicles
MSC-Ex	Mesenchymal stem cell-derived exosomes
mtDNA	Mitochondrial DNA
MVB	Multivesicular body
NAFLD	Non-alcoholic fatty liver disease
Nrf2	Nuclear factor erythroid 2-related factor 2
NTA	Nanoparticle tracking analysis
OMVs	Outer membrane vesicles
PA	Phosphatidic acid
PC	Phosphatidylcholine
PDGF-BB	Platelet-derived growth factor-BB
PDNVs	Plant-derived nanovesicles
PEG	Polyethylene glycol
PELNs	Plant-derived exosome-like nanoparticles
PEVs	Plant extracellular vesicles
PLNVs	Plant lipid-derived nanovesicles
PS	Phosphatidylserine
ROS	Reactive oxygen species
SEC	Size exclusion chromatography
SOD	Superoxide dismutase
TAMs	Tumor-associated macrophages
TEM	Transmission electron microscopy
TET8	Tetraspanin 8
TET9	Tetraspanin 9
TGN	Trans-Golgi network
TNF-α	Tumor necrosis factor-α
UC	Ulcerative colitis
UF	Ultrafiltration
VH	Viral hepatitis
ZIF-8	Zeolitic imidazolate framework-8
